# A relationship between oxytocin and anxiety of romantic attachment

**DOI:** 10.1186/1745-0179-2-28

**Published:** 2006-10-11

**Authors:** Donatella Marazziti, Bernardo Dell'Osso, Stefano Baroni, Francesco Mungai, Mario Catena, Paola Rucci, Francesco Albanese, Gino Giannaccini, Laura Betti, Laura Fabbrini, Paola Italiani, Alessandro Del Debbio, Antonio Lucacchini, Liliana Dell'Osso

**Affiliations:** 1 Dipartimento di Psichiatria, Neurobiologia, Farmacologia e Biotecnologie, University of Pisa, Italy; 2 Compulsive, Impulsive and Anxiety Disorders Program, Department of Psychiatry, Mount Sinai School of Medicine, New York, USA

## Abstract

The formation of social bonding is fundamental for several animals, including humans, for its relevant and obvious impact upon reproduction and, thus, survival of the species. Recent data would suggest that oxytocin might be one of the mediators of this process. Given the paucity of data on the possible involvement of oxytocin in human attachment, the present study was aimed to explore the possible relationships between the plasma levels of this neuropeptide and romantic attachment in healthy subjects. Forty-five healthy subjects who volunteered for the study, were included in the study. The romantic attachment was assessed using the Italian version of the so-called "Experiences in Close Relationships" (ECR), a self-report questionnaire for measuring this parameter in adults. The results showed that attachment anxiety and oxytocin are positively linked in romantic attachment to a statistically significant degree (r = 0.30, p = 0.04), that is, the higher the oxytocin levels the higher the score on the anxiety scale of the ECR. The authors suggest the hypothesis that this link represents one of the biological processes resulting in those rewarding emotions related to romantic attachment.

## Background

Humans are obliged to face a paradox which is fundamental to the survival of the species: they are attracted to, courted by and breed with genetically unrelated individuals whom they would otherwise instinctively avoid. Romantic attachment is the psychological strategy which enables us to overcome neophobia and to mate with and create a strong, often life-long bond with a complete stranger, so that we may produce healthier offspring. On the same time, we are rewarded by a deep sense of pleasure and satisfaction through the intervention of specific neural substrates which have become the topic of recent investigations [1]. Besides neural networks, within the last decade, various sets of data have highlighted the possible role of oxytocin and vasopressin, two small neuropeptides composed by 9 aminoacids and synthesized in the paraventricular and in the supraoptic nuclei of the hypothalamus [2], in linking social signals with cognition, behaviours and reward [3]. Oxytocin and vasopressin, in fact, have been shown to be involved in the creation of pair-bonding in monogamous rodents, such as prairie voles [4], in maternal behaviour in rats [5-7], in the postpartum acceptance of offspring in sheep [8,9], and the relief of distress vocalization in rat pups [10].

The relevance of these intriguing findings for humans has not been clarified as yet [11,12]. Oxytocin receptors in the human brain are mainly distributed in the substantia nigra, globus pallidus, anterior cingulate and medial insula, areas which have been shown to be activated in adults while looking at pictures of their partners, or in mothers while looking at their children [1,13,14] and belong to the recently-hypothesized circuits of the "social brain" [15-17]. It is interesting to note that oxytocin seems to be released during sexual intercourse and orgasm [18] and during the application of different relaxation techniques [19,20], so that it is thought to be one of the promoters of attachment and/or mediators in the decrease of the stress responses which are related to positive social bonding [21-23].

Although the measurement of oxytocin plasma levels represents the easiest and relatively noninvasive way of evaluating its presence in humans, it is still unclear whether plasma levels reliably reflect concentrations in the brain, since it would seem that central and peripheral oxytocin derive from different hypothalamus cell populations [4,9]. However, different convergent observations on parallel changes in oxytocin levels in plasma and in the brain [20,24,25] would suggest a cooperative process of regulation and, thus, support the use of the peripheral marker as a mirror of the central one [8,26]. Therefore, given the lack of direct data in humans, in the present study we explored the possible relationships between plasma oxytocin levels and a typical social bonding of our species, that is, romantic attachment, selected from amongst the different types of attachment, because it was considered particularly relevant for adults that constituted our subject population [27]. In addition, to achieve this goal, we had to set up a sensitive assay to measure oxytocin in plasma of healthy subjects, since the available kits did not permit this assessment; the hypothesis was that oxytocin might influence some features or styles of romantic attachment.

## Materials and methods

### Subjects

Forty-five healthy subjects (12 men, 33 women, mean age 31.5 ± 6.2 years) all of whom volunteered for the study, were included. They were residents, post-doctoral fellows or clinicians at the Specialty School of Psychiatry, or students at the Faculty of Medicine and Surgery, all at the University of Pisa. In terms of marital status, 32 (71.1 %) individuals were single, 12 (26.7 %) were married and 1 (2.2 %) was divorced.

No subject had a family or personal history of any major psychiatric disorder, or had ever taken regularly psychotropic drugs, as assessed by a detailed psychiatric interview conducted by one of the authors (DM). All subjects were free of physical illness, were neither heavy cigarette smokers, nor belonged to groups of high-risk HIV individuals, nor did take any regular medication or drugs of addiction. The women had normal menstrual cycles and did not take contraceptive pills; their blood was drawn in the early follicular phase (between the second and the fifth day of the menses). The men had no history of genital disease or hypogonadism. All these information derived from the medical history collected by two authors (FM, MC).

Thirty-three subjects had a current romantic relationship with a mean duration of 80.5 months (ranging from a minimum of one month to a maximum of 25 years); the remaining 12 had no current relationship.

Prior to enrolment, participating subjects gave their written informed consent to the study which was approved by the Ethics Committee of the University of Pisa.

### Instruments

The romantic attachment was assessed using the Italian version of the so-called "Experiences in Close Relationships" (ECR) [28], a self-report questionnaire for measuring this parameter in adults. The Italian version has proven to have good psychometric properties of validity and reliability [29]. It consists of 36 items, scored on a seven-point Likert scale, with 1 indicating "completely false" and 7 indicating "completely true". In addition, it provides two scale scores measuring anxiety and avoidance, the two main dimensions underlying adult attachment styles. According to the scores on these scales, subjects can be classified in terms of 4 mutually exclusive categories of attachment. The age- and sex-stratified norms obtained in the validation study of the Italian version were used. Scores within one standard deviation from the norm were considered as normal. Participants scoring above normal on the anxiety scale were classified as preoccupied, those scoring above normal on the avoidance scale were classified as dismissing, those scoring above normal on both scales were classified as fearful/avoidant, while all the remaining participants were classified as secure.

### Methods

In the months of January and February, and between 8 and 9 am, blood (20 cc) was drawn three times from fasting subjects who were sitting and relaxing in the same room at a constant temperature. Blood was collected in vacutainers containing EDTA as anticoagulant, transferred to centrifuge tubes containing aprotinin (Sigma, Milan, Italy) (0.6 TIU/ml of blood) and gently mixed several times to inhibit the activity of proteinases. Blood was then centrifuged at 1,600 × g for 15 minutes at 4°C and the ensuing plasma was collected and kept at -70°C until the assay.

Four subjects (2 M, 2 F) underwent repetitive blood samplings (n. 18) on the same day, under the same experimental conditions in order to ascertain the possible presence of a circadian rhythm with oxytocin which, however, would seem to be absent (data not shown), as already reported [30,31].

### Extraction of peptides from plasma

On the day of the assay, 6 ml of each sample of plasma was acidified with 6 ml of buffer A (1% trifluoroacetic acid in H_2_O) and centrifuged at 17,000 × g for 20 minutes at 4°C; after this centrifugation, the supernatant was collected. C-18 sep-columns were equilibrated by washing them with 1 ml of buffer B (60% acetonitrile in 1% trifluoroacetic acid) followed by buffer A (3 ml, 3 times). Acidified plasma solution was loaded into the pre-treated C-18 Sep-column; the column was washed slowly with buffer A (3 ml, twice) and the washing liquid was discarded. Oxytocin was then eluted with buffer B (3 ml, once) and collected into a polystyrene tube. The eluate was evaporated in a centrifugal concentrator (Speedvac) and the remaining sample was lyophilized by freeze dryer.

### Oxytocin radioimmunoassay

Radioimmunoassay was performed by using Phoenix Pharmaceuticals Oxytocin RIA kit (Belmont, California, USA), with a method developed by us. The cross-reactivity of the oxytocin antibody was 100% with oxytocin and O with Lys-vasopressin, Arg-vasopressin, GH, alpha-ANP, Met-Enkephalin, GRF, somatostatin, TRH, VIP, Pacap 27-NH_2_. The sensitivity of the assay, measured as IC_50_, was 10–30 pg/tube. The intrassay and interassay values were 9% and 11%, respectively.

Lyophilized samples and standard oxytocin were re-hydrated with RIA buffer, and dilutions of standard oxytocin were prepared (from 1 to 128 pg/tube). Primary rabbit anti-oxytocin antibodies were added to each sample and each standard, except for the non-specific binding tubes, and then the mixtures were stored for 24 hours at 4°C. ^125^I-Oxytocin was added to mixtures which were subsequently stored for 24 hours at 4°C. Goat anti-rabbit IgG serum and normal rabbit serum were added to each tube; subsequently, tubes were centrifuged at 1700 × g for 20 minutes at 4°C. All the supernatant was carefully aspirated and pellets were counted by a gamma-counter (Wizard, Perkin Elmer, Milan, Italy).

All samples were assayed in duplicate.

Standard curve and calculations of unknown samples were performed by using Graphpad Prism3 software via personal computer programmes.

### Statistical analyses

Correlations between the levels of oxytocin and the duration, presence/absence of a romantic relationship, the anxiety or avoidance scales of the ECR and the styles of attachment, as well as the demographic characteristics of the subjects, were examined using Pearson's correlation coefficient.

Oxytocin levels were compared between genders and between subjects with and without a romantic relationship, using the *t*-test (two-tailed, unpaired), and between subjects with different attachment styles with ANOVA.

All analyses were carried out using SPSS, version 12.01 (2003).

## Results

The oxytocin plasma levels, representing the mean+SD of three evaluations performed within one hour, ranged between 0.13 ± 0.02 and 4.59 ± 0.01 pg/ml (mean ± SD: 1.53 ± 1.18), and the frequency distribution of maximum oxytocin levels was skewed to the right. No difference was detected between women and men, or between subjects with and without a romantic relationship.

**Figure 1:**
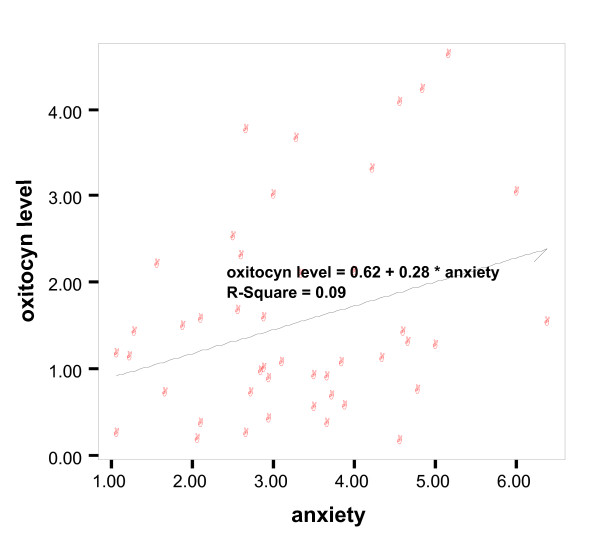
The relationship between oxytocin palsma levels and the anxiety scale total score of the ECR.

Plasma oxytocin levels were unrelated with age, gender, marital status, or length of the relationship, while, as shown in figure 1, a significant and positive correlation was observed between the anxiety scale of the ECR and oxytocin levels (r = 0.30, p = 0.04); on the contrary, no relationship with the avoidance scale was detected.

The distribution of attachment styles was typical of healthy control subjects. Twenty-six (57.8 %) subjects showed a secure attachment, 12 (26.7 %) a preoccupied, 5 (11.1) a fearful/avoidant and 2 (4.4 %) a dismissing style. A trend towards higher peptide concentrations was detected in those subjects with the preoccupied style of attachment, although it did not reach the statistical significance.

## Discussion

The major finding of the present study was the detection of a statistically significant and positive correlation between oxytocin plasma levels and the anxiety scale of the ECR, a self-report questionnaire measuring adult romantic attachment, which showed that the higher the oxytocin levels, the higher the score on the anxiety scale of the ECR.

Some caution is, however, warranted in the interpretation of these results, given the controversies which exist regarding the reliability of plasma oxytocin levels as a peripheral mirror of central concentrations. In any case, it is worth noting that various physiological conditions, in particular labour, lactation and sexual activity, have been shown to provoke a parallel release of central and peripheral oxytocin [8,9], that emotional states may sometimes modify plasma oxytocin [20,25,32], and that even the peripheral administration of different peptides, including cholecystokinin and interleukin 6, may trigger their central release [26].

In addition, oxytocin shows a pulsatile secretion, so that, in order to overcome this possible bias, all subjects included in our study underwent three blood samplings within one hour and the analyses were performed by using the means of the different measurements.

Another bias may be related to the subjects include in the study, all belonging to the medical class, so that our sample may not be representative of the general population,

In spite of the above-mentioned limitations, our findings may constitute undoubtedly the first report of a link between oxytocin and that state of anxiety which is associated with romantic attachment in our species. However, it is not possible to conclude from our data whether the oxytocin levels are in fact a consequence or a cause of the anxiety measured by the scale of the ECR, although, in line with the majority of available findings showing in animals anxiolytic-like properties for oxytocin [33-35], we would suggest tentatively that the former might be the case, and that oxytocin might serve to help to counteract anxiety – or, at least, that form of anxious stress associated with romantic attachment and deep concern over its continuance [11,19,21,36].

Previoulsy, the relationship between oxytocin and human anxiety was sustained only by the indirect evidence that basal oxytocin levels correlate with measures of anxiety, aggression, guilt and suspicion [37], and noise stress provoke the release of the neuropeptide in highly emotional women [38]. Also, recent findings have reported that low plasma oxytocin levels would seem to be typical of individuals with low anxiety traits [39].

That oxytocin and anxiety may be linked in some way in the modulation of social bonding is supported also by scattered data showing that a moderate level of stress seems to promote pair bonding in different species, including human beings [40]. Pursuing this line of thought, romantic relationships, and perhaps social relationships in general, could be interpreted as amounting to stress conditions, both acute and chronic depending on the phase [41]. The role of oxytocin would seem generally to be that of keeping anxiety levels under control to a point where they are no longer harmful (in fact, low oxytocin concentrations have been linked with pain syndromes, such as fibromyalgia [42] or abdominal pain [43], but may nevertheless lead to such strategies and behaviours as are best suited to ensure a partner's continued proximity both during the first stages of the romance and subsequently. Oxytocin might thus be considered an essential element in securing the rewarding effects of a romantic relationship, as a result of its increasing a prospective sexual partner's willingness to accept the risk deriving from social contacts [23], through the modulation of anxiety mechanisms perhaps at the level of amygdala [33].

Of course, with particularly vulnerable individuals, if excessively affected by the relationship itself or by other events, these delicate mechanisms might be maladaptive, in the sense that such subjects might become too anxious and thus cross the line between normal and pathological states, even to the point of developing a full-blown psychiatric disorder [44]. Such a hypothesis might be relevant to the onset of anxiety disorders and disturbances of social communications, such as autism.
